# Quantification of Lung Perfusion Blood Volume by Dual-Energy CT in Patients With and Without Chronic Obstructive Pulmonary Disease

**DOI:** 10.5334/jbr-btr.865

**Published:** 2015-09-15

**Authors:** H. Koike, E. Sueyoshi, I. Sakamoto, M. Uetani

**Affiliations:** 1 Department of Radiology, Nagasaki University School of Medicine, Sakamoto, Nagasaki, Japan

**Keywords:** Lung, perfusion – Subtraction, dual energy – Pulmonary arteries, stenosis or obstructive

## Abstract

**Purpose:** In chronic obstructive pulmonary disease (COPD), pulmonary vascular alteration is one of the characteristic features. Recently, software has been used for the quantification of lung iodine perfusion blood volume (iPBV) using dual-energy CT, allowing objective evaluation. The purpose of this study was to evaluate the quantification of lung PBV with and without COPD.

**Materials and Methods:** This study was approved by the Institutional Review Board. Sixty-two subjects who had undergone a respiratory function test within one month underwent dual-energy CT angiography. The subjects were divided into two groups: with (n = 14) and without (n = 48) COPD. We evaluated the quantification of lung iPBV in the early phase and late phase using Syngo softwarepost contrast. Associations between lung iPBV and respiratory function (forced expiratory volume in 1 second/forced vital capacity; FEV1/FVC) and the percentage area of emphysema (%LAA-950) were also evaluated.

**Results:** In the early phase, lung iPBV values were 20.1 ± 5.5 and 30.6 ± 7.6 Hounsfield Unit (HU) in those with and without COPD, respectively, with a significant difference between them (p < 0.0001). In the late phase, the values were 12.3 ± 3.7 and 15.3 ± 4.6 HU, respectively, with no significant difference (p = 0.051). However, this could be noticed as a trend. In the early phase, there was a weak significant correlation between lung iPBV value and FEV_1_/FVC (R = 0.26, p = 0.047). There were significant and moderate negative correlations between lung iPBV value and %LAA-950 in early and late phases (R = −0.57, p = 0.0002; R = −0.45, p = 0.005, respectively).

**Conclusions:** Quantification of lung iPBV reflects reduced pulmonary perfusion in patients with COPD. It may be useful for objective evaluation of the pulmonary blood flow in patients with COPD.

The introduction of a dual-source CT system in the same gantry resolved many of the technical issues that previously limited the application of dual-energy CT pulmonary angiography. Dual-energy CT pulmonary angiography does not expose patients to any significant additional radiation beyond that of standard CT pulmonary angiography [1, 2]. Post-processing software is then used to subtract 80-kV from 140-kV images to produce an iodine distribution map image (lung iodine perfusion blood volume image). Lung iodine perfusion blood volume (iPBV) imaging using dual-energy CT enables the creation of iodine maps of the pulmonary parenchyma. Experience to date has shown that these studies can provide additional physiological information on patients with acute or chronic pulmonary embolism (PE) beyond the purely morphological assessment that standard CT pulmonary angiography provides [3, 4].

Recently, software (Syngo MultiModality; Siemens Healthcare) has been used for the quantification of lung iodine perfusion blood volume (iPBV), allowing objective evaluation. In addition, compared with visual analysis of PBV images, a quantitative technique can confer an advantage in avoiding any interobserver variance due to the variety of image display parameters available for dual-energy CT iPBV images. Additionally, this method, with its automatic quantification, is rapid and simple to use.

Several reports have shown that perfusion reduction corresponds to air cyst or emphysema because of reduction of the pulmonary parenchyma [5, 6]. However, few reports have shown the objective quantification of lung iPBV correlating with COPD.

The purpose of this study was to evaluate the objective quantification of lung iPBV with and without COPD using Syngo software.

## Materials and methods

### Patient population

This study was approved by the Institutional Review Board. Five hundred and forty-one patients underwent dual-energy pulmonary CT because of suspected pulmonary embolism between May 2009 and December 2012 at our hospital. They underwent dual-energy pulmonary CT because of suspected pulmonary embolism. Patients with pulmonary thromboembolism were excluded by CT findings in this study. We also excluded from our study group those patients who had positive D-dimer, lung disease including pneumonia, lung tumor, cardiac disease, and pleural effusion. Finally, we retrospectively evaluated 62 patients (21 men, 41 women; mean age, 68.9 ± 15 years) who had undergone a respiratory function test within one month.

### CT acquisition protocol

All patients were examined with a dual-source CT scanner (Somatom Definition; Siemens Healthcare) in dual-energy mode. At first, precontrast CT of the chest images were obtained. After that, the examinations were performed with an adapted contrast injection protocol aimed at displaying both angiograms of the pulmonary arteries and parenchymal iodine distribution. Injections were administered through a 20-gauge cannula in an antecubital vein. High-concentration iodine-based contrast material (Omnipaque 350; Daiichi-Sankyo, Tokyo) was administered at a flow rate of 4.0 ml/s followed by a 40-ml saline chaser bolus at the same injection rate. The total volume of contrast material was adapted to the patient’s body weight at 1.35 ml/kg. In this study, CT scan began at a fixed 14 s from the start of injection in the early phase and was taken again at 40 s from the start in the late phase. In our facility, the data of late phase are usually obtained to evaluate parenchymal distribution of iodine. To avoid streak artifacts due to highly concentrated contrast material in the subclavian vein or superior vena cava, scans were acquired in the caudocranial direction so that the chaser bolus was being injected by the time the scan reached the upper chest. Other scan parameters were as follows: tube voltage, 140 and 80 kVp at 30 and 210 effective mAs; attenuation-based tube current modulation; rotation time, 0.5 s; collimation, 14 × 1.2 mm; and pitch, 0.7. The average volume CT dose index was 7.0 mGy/cm, resulting in an average equivalent dose of 3.0 mSv, covering the whole chest. CT angiography (CTA) images were reconstructed with a specific medium-soft convolution kernel (D30) without edge modification at 1.0-mm slice thickness with a 1.0-mm increment. The image reconstruction system generated three stacks of axial images, that is, 140-kV images, 80-kV images, and weighted-average images that derived 60% of image density from the 140-kV images and 40% from the 80-kV images to achieve image quality and a signal-to-noise ratio similar to that of single-energy 120-kV scans.

### Pulmonary function tests

Postbronchodilator spirometry was performed according to American Thoracic Society standards. Forced vital capacity (FVC) and forced expiratory volume in 1 second (FEV1) were measured.

### Quantitative measures of emphysema

On precontrast CT, quantitative measures of emphysema for the whole lung were calculated using software (Zio station2). Emphysema was defined as low-attenuation areas using a Hounsfield unit (HU) threshold of −950 (%LAA-950). All patients with COPD were classified in terms of Global Initiative for Chronic Obstructive Lung Disease (GOLD) stage.

### CTA data

CTA data were assessed by two experienced radiologists (with over 10 years of experience in cardiothoracic CT) who were blinded and worked independently. Axial CTA data (soft tissue window setting) at 1.0-mm slice thickness with a 1.0-mm increment were reviewed by them. Multiplanar reformatted (coronal) images at 1.0-mm slice thickness with a 1.0-mm increment were also evaluated for the presence of other lung diseases in order to be excluded from our study. In the case of different findings, the data set was reviewed in order to reach a consensus.

### Analysis of dual-energy data

**Figure 1:**
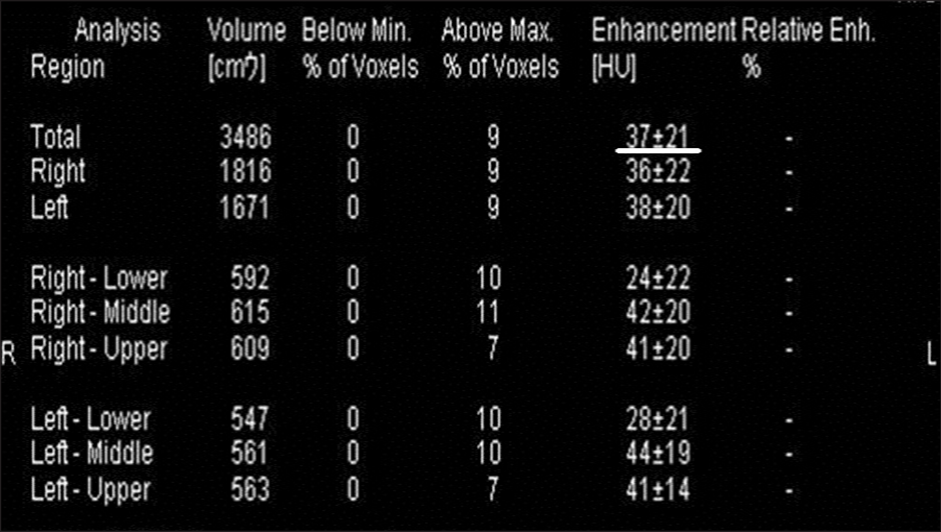
Hounsfield units (HU) (iodine enhancement level) as lung iPBV values are calculated automatically in several patterns, including the whole lung (underlined).

On the basis of three-material decomposition of soft tissue, air, and iodine, iodine distribution maps of the lungs were generated on a workstation (Syngo MultiModality; Siemens Healthcare) with specific dual-energy post-processing software. Hounsfield units (HU) were automatically calculated in several patterns, including both lungs, and right or left lungs (Fig. 1). We used the data of both lungs in this study.

### Statistical analysis

All values are expressed as the mean ± SD. Statistical analysis was performed using clinical and morphological variables with the paired t test and Mann–Whitney’s U-test for continuous variables. The results are expressed as the sensitivity, specificity, and overall accuracy, with 95% confidence intervals (CIs) calculated with the normal approximation method [7]. Pearson correlation coefficients were used to examine correlations. We created receiver operating characteristic (ROC) curves and determined the threshold that led to the optimal values of probabilities in the presence or absence of PE. This optimal threshold was defined as the intersection of the ROC curve with the bisecting line at which sensitivity equaled specificity. Correlation coefficient values of 0.4–1.0 were considered to indicate a correlation [8]. In all tests, p < 0.05 was considered significant (release 11.5; SPSS, Chicago, IL).

## Results

### Analysis of patients with and without COPD

**Figure 2:**
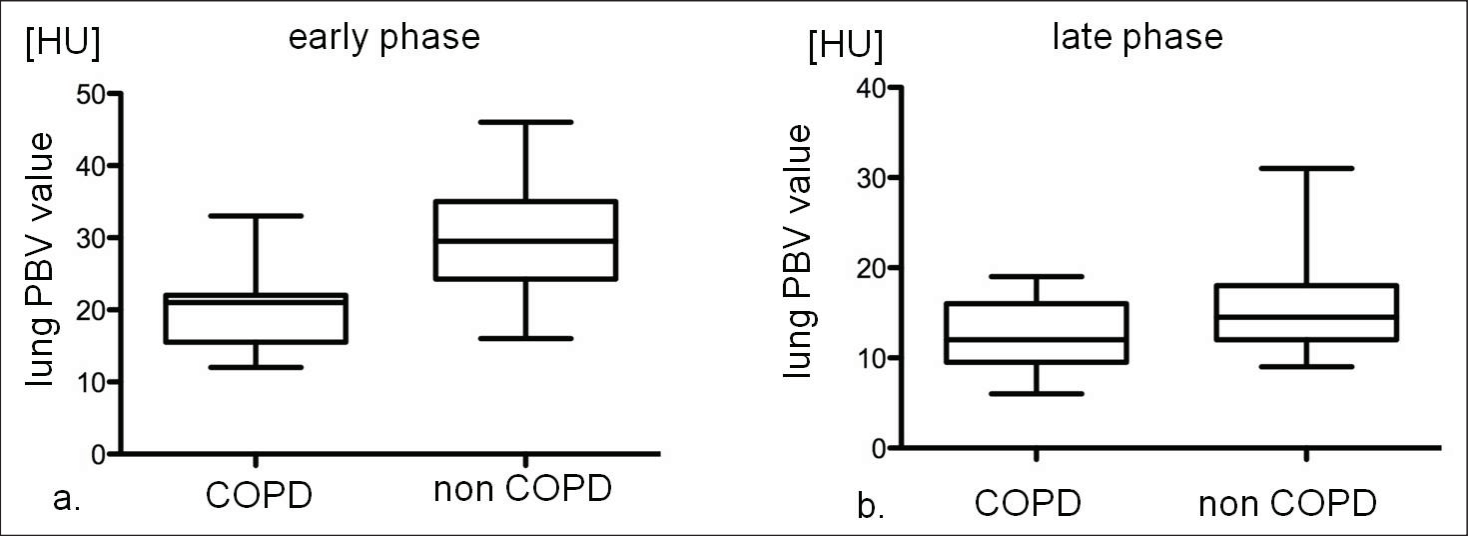
Individual data of lung iPBV value with or without COPD: in early phase (a) and in late phase (b).

Table 1 shows a summary of the results. In total, 14 of 62 (23%) patients had COPD and 48 (77%) did not. All patients with COPD were classified into GOLD stage 1 or 2 (GOLD 1: 13, 2: 1), and they had mild emphysema. For patient (whole lung)-based analysis in the early phase, lung PBV values were 20.1 ± 5.5 and 30.6 ± 7.6 HU in patients with and without COPD, respectively. There was a significant difference between these two groups (p < 0.0001). In the late phase, lung PBV values were 12.3 ± 3.7 and 15.3 ± 4.6 HU in patients with and without COPD, respectively. There was no significant difference between these two groups (p = 0.051) (Fig. 2). However, this could be noticed as a trend. There were moderate correlations in lung iPBV values between early and late phases (R = 0.58, p < 0.0001).

**Table 1:** Summary of results of analysis with and without COPD. Note: NS = not significant. FEV_1_/FVC = forced expiratory volume in 1 second/ forced vital capacity. %LAA-950 = low attenuation areas using a Hounsfield Unit (HU) threshold of −950.

	Patients with COPD(n = 14)	Patients without COPD(n = 48)	p value

Age(years)	70.4 ± 10.3	65.3 ± 15.3	NS
Male,n	8	13	NS
Lung iPBV in early phase (HU)	20.1 ± 5.5	30.6 ± 7.6	< 0.001
Lung iPBV in late phase (HU)	12.3 ± 3.7	15.3 ± 4.6	0.051
FEV_1_/FVC(%)	63.1 ± 5.4	79.1 ± 7.1	< 0.001
%LAA-950(%)	8.0 ± 3.8	4.0 ± 2.4	< 0.001

**Figure 3:**
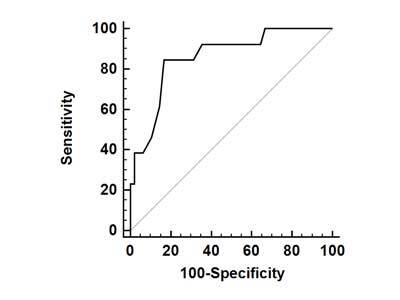
ROC analyses demonstrated moderate discriminatory power for using lung iPBV to differentiate between patients with and without COPD. When less than 27.5 HU was used as the threshold for diagnosis, the sensitivity, specificity, positive predictive value, and negative predictive value were 92.3, 64.6, 41.4, and 96.8%, respectively.

ROC analyses (Fig. 3) demonstrated moderate discriminatory power for using the quantification of lung PBV to differentiate between patients with and without COPD in the early phase. When less than 27.5 HU was used as the threshold for diagnosis, the sensitivity, specificity, positive predictive value, and negative predictive value were 92.3, 64.6, 41.4, and 96.8%, respectively.

### Associations with respiratory function (FEV_1_/FVC)

In the early phase, there was a significant correlation between lung iPBV and respiratory function (FEV_1_/FVC) (R = 0.26, p = 0.047). In the late phase, there was no significant correlation between lung PBV value and respiratory function (FEV_1_/FVC) (R = 0.05, p = 0.70).

### Associations with percentage area of emphysema (%LAA-950)

**Figure 4:**
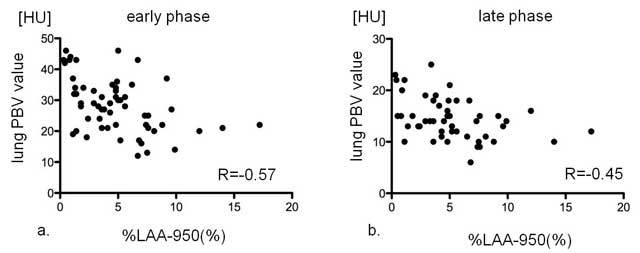
The relationship between lung iPBV value and the percentage area of emphysema (%LAA-950) in all patients: in early phase (a) and in late phase (b). The lung iPBV values in early phase (R = −0.57, p = 0.0002) and late phase (R = −0.45, p = 0.005) had significant negative correlations with %LAA-950.

There were moderate correlations between lung iPBV value and the percentage area of emphysema (%LAA-950) in early and late phases (R = −0.57, p = 0.0002; R = −0.45, p = 0.005, respectively) (Fig. 4).

## Discussion

Lung iPBV imaging using dual-energy CT (DECT) has recently become available on clinical CT systems. The underlying physical principle of DECT is the fact that the photoelectric effect is strongly dependent on the CT energy, resulting in different degrees of X-ray attenuation for different materials at different energy levels. DECT thus enables the characterization and quantification of iodine within tissues via imaging at different X-ray energies and analysis of attenuation differences. Technical approaches to DECT include dual-source scanners acquiring two scans with different energy levels simultaneously, and single-source CT scanners using sandwich detectors or rapid voltage switching. Lung iPBV imaging using dual-energy CT enables the creation of iodine maps of the pulmonary parenchyma. Experience to date shows that these studies can provide additional physiological information in patients with acute or chronic pulmonary embolism beyond the purely morphological assessment that standard CT pulmonary angiography (CTPA) provides [3, 4, 8,9,10,11,12,13,14,15].

It also appears to be promising for the evaluation of patients with obstructive airway disease [16,17,18]. Recently, the degree of decrease of perfusion in lung PBV has been shown to be correlated with the severity of emphysema [13, 19].

However, there have been few reports which objectively evaluated the relationship between the quantification of lung PBV and COPD using Syngo software, which is only used for Siemens CT scanner.

According to previous reports, pulmonary vascular alteration is a characteristic feature of COPD. Early angiographic studies in patients with emphysema showed narrowing and reduction in the number of small pulmonary arteries at subsegmental or sub-subsegmental levels [20,21,22]. Passive vascular compression by emphysema and hypoxic vasoconstriction has been considered the major pathogenesis of vascular alteration in COPD. Histologically, pulmonary vascular alterations are not exclusive to advanced COPD, and they are present in patients with mild COPD and even in smokers with normal pulmonary function [5, 6, 23,24,25,26,27]. Recent studies have suggested that both pulmonary and extrapulmonary vascular alterations in patients with COPD are closely related to endothelial dysfunction [27,28,29]. Because of the important role played by the endothelium in regulating vascular tone and controlling cell growth, pulmonary arteries with endothelial dysfunction have a diminished ability to dilate [30]. Noma et al [31] experimentally showed a decrease in pulmonary perfusion in mild emphysema without significant ventilation abnormality. Together, these studies suggest that lung pulmonary blood volume in COPD is not always associated with ventilation impairment. Consequently, pulmonary parenchyma destruction occurs and leads to functional impairment.

In this study, we found that there was a significant difference in lung iPBV values between patients with and without COPD in the early phase. Meanwhile, there was no significant difference in the late phase. We speculate that lung iPBV value in the late phase mainly reflects reduction of pulmonary parenchyma; on the other hand, in the early phase it mainly reflects vascular alteration. In the early phase, pulmonary artery blood flow is shifting to the pulmonary parenchyma, but this shift has not yet been completed. In the late phase, pulmonary artery blood flow has already shifted to pulmonary parenchyma. Therefore, in the late phase, lung iPBV value might mainly reflect reduction of pulmonary parenchyma. In contrast, in the early phase, this value might reflect the reduced pulmonary perfusion due to vascular alteration more strongly than in the late phase. This study showed not so high correlations in lung iPBV values between early and late phases, which may support this speculation.

In this study, there was no significant difference in lung iPBV values between patients with and without COPD in the late phase. All patients with COPD were classified into GOLD stage 1 or 2 (GOLD 1: 13, 2: 1), and they had mild emphysema. Therefore, we speculate that they had mild reduction of lung parenchyma, which might have led to these results.

Lung iPBV value correlated with %LAA in the early phase and the late phase (R = −0.57, p = 0.0002; R = −0.45, p = 0.005, respectively) in all patients. Meanwhile, correlations of lung PBV value with FEV1/FVC were significant but weak in the early phase, and there were no significant differences in the late phase in all patients. These results might indicate that pulmonary parenchyma destruction occurs earlier than functional impairment, which supports the findings of previous studies [23,24,25].

### Clinical implications

Pulmonary vascular alteration is a characteristic feature of COPD. However, there have been no methods available for the quantification of pulmonary parenchyma perfusion itself. Lung iPBV may be a useful tool for its quantification in COPD. Additionally, lung iPBV, CT, and CTA images can be obtained at the same time. Therefore, the other serious causes of dyspnea including pulmonary embolism can be excluded at the same time. As a result, lung iPBV images can reduce radiation exposure in patients.

In view of the above benefits, this method might be a useful tool for the evaluation of mild COPD. With regard to patients with severe COPD, evaluation by respiratory function test is often difficult. This method can be evaluated objectively and may also be a useful tool for the evaluation of severe COPD. Additionally, this method, with its automatic quantification, is rapid and simple to use.

### Limitations

There were several limitations in this study. First, it included a limited number of patients, and almost all patients with COPD had mild emphysema. In this study, they underwent dual-energy pulmonary CT because of suspected pulmonary embolism. Patients with pulmonary thromboembolism were excluded by CT findings in this study. Therefore, a limited number of patients was evaluated. Our data constitute preliminary results and further studies are needed.

A second limitation was patient selection. The patients with pulmonary thromboembolism were excluded by CT findings. We also excluded patients who had positive D-dimer, lung disease including pneumonia, lung tumor, cardiac disease, and pleural effusion. However, there was subject selection bias. The third limitation was that coverage of the whole lung volume within this reduced FOV is not feasible. In these cases (20 of 62 cases), small peripheral defects might have been missed. For the best possible solution to this problem, the patient has to be positioned exactly in the center of the gantry to ensure that most of the lung is inside the area covered by both tubes, which is essential for dual-energy imaging.

## Conclusions

Quantification of lung iPBV reflects reduced pulmonary perfusion and correlates with respiratory function (FEV_1_/FVC) and %LAA-950 in patients with COPD. It may be useful for objective evaluation of the pulmonary blood flow in patients with COPD. This method can be a new diagnostic tool for the evaluation of COPD; however, further studies are needed.

## Competing Interests

The authors declare that they have no competing interests.
